# Development and validation of a portfolio assessment system for medical schools in Korea

**DOI:** 10.3352/jeehp.2020.17.39

**Published:** 2020-12-09

**Authors:** Dong Mi Yoo, A Ra Cho, Sun Kim

**Affiliations:** Department of Medical Education, College of Medicine, The Catholic University of Korea, Seoul, Korea; Hallym University, Korea

**Keywords:** Medical education, Portfolio, Assessment, Republic of Korea

## Abstract

**Purpose:**

Consistent evaluation procedures based on objective and rational standards are essential for the sustainability of portfolio-based education, which has been widely introduced in medical education. We aimed to develop and implement a portfolio assessment system, and to assess its validity and reliability.

**Methods:**

We developed a portfolio assessment system from March 2019 to August 2019 and confirmed its content validity through expert assessment by an expert group comprising 2 medical education specialists, 2 professors involved in education at medical school, and a professor of basic medical science. Six trained assessors conducted 2 rounds of evaluation of 7 randomly selected portfolios for the “Self-Development and Portfolio II” course from January 2020 to July 2020. These data are used inter-rater reliability was evaluated using intra-class correlation coefficients (ICCs) in September 2020.

**Results:**

The portfolio assessment system is based on the following process; assessor selection, training, analytical/comprehensive evaluation, and consensus. Appropriately trained assessors evaluated portfolios based on specific assessment criteria and a rubric for assigning points. In the analysis of inter-rater reliability, the first round of evaluation grades was submitted, and all assessment areas except “goal-setting” showed a high ICC of 0.81 or higher. After the first round of assessment, we attempted to standardize objective assessment procedures. As a result, all components of the assessments showed close correlations, with ICCs of 0.81 or higher.

**Conclusion:**

We confirmed that when assessors with an appropriate training conduct portfolio assessment based on specified standards through a systematic procedure, the results are reliable.

## Introduction

### Background/rationale

A portfolio refers to a learner’s collection of evidence supporting his or her educational trajectory, as well as records of reflections on his or her progress and achievements. Portfolio-based assessment is a comprehensive and holistic method of evaluation that provides a concrete basis for growth in expertise, knowledge, technical aptitude, and understanding through the learner’s self-reflection. It is among the most favored approaches to performance evaluation, which is a framework that emphasizes comprehensive and regular evaluations, as opposed to series of one-time assessments dealing with confined segments of the curriculum, in order to comprehensively assess the individual learner’s processes of change and development [1,2]. Portfolio assessment brings about a closer association between the assessment process and learning, and allows the assessor to confirm the extent of a learner’s progress by providing feedback. Moreover, portfolio assessment is more efficient than conventional methods for evaluating students’ progress in terms of attitudes, personal qualities, and professional ethics, which are difficult to assess using traditional means. Due to these advantages, portfolio assessment has recently emerged as a focus of attention in medical education [3]. However, establishing a consistent and stable system based on objective and reasonable assessment standards is essential for portfolio-based assessment to be operated as a longitudinal program within the framework of the regular curriculum. If the quality management of assessment tools and procedures becomes less rigorous due to an excessive emphasis on the positive role of portfolio assessment for its own sake, the ability of the system to determine crucial aspects of a learner’s ability would be limited [4]. Most notably, the accountability of the assessor should be addressed. Since the reliability of performance evaluation systems, such as portfolios, depends on the assessor’s observations of the performance and outcomes of the person being assessed, inter-observer and intra-observer reliability are considered as more important factors than the reliability of the instrument itself. The problem of whether one can trust the result of the performance evaluation process normally comes down to how consistent or reliable the assessors are—or, in other words, the issue of inter-rater reliability [5]. Therefore, reducing inter-rater discrepancies in evaluation is crucial for ensuring the reliability of the assessment procedure.

### Objectives

To evaluate the validity and reliability of the portfolio assessment procedure, we established the following research objectives. First, we developed a portfolio assessment system to implement in the “Self-Development and Portfolio” course within the regular curriculum of College of Medicine, the Catholic University of Korea. Second, we verified the validity of the portfolio assessment system through content validity analysis by experts, and conducted an analysis of inter-rater reliability.

## Methods

### Ethics statement

This study was approved by the Institutional Review Board of Songeui Medical Campus, the Catholic University of Korea (IRB approval no., MC20EISI0122). No informed consent forms were collected, but the participants were clearly informed of the purpose of this study and were not pressured to participate in any way. Therefore, there were no disadvantages to non-participation. A waiver of consent was also included in the IRB approval.

### Study design

It is a psychometric study for the validity and reliability test of the measurement tool.

### Setting

**Fig. 1.**
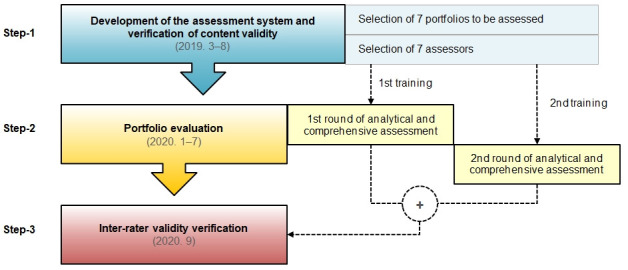
Diagram of the research process.

This study involved 3 steps, as outlined in detail in Fig. 1.

### Development of the assessment system

We developed a portfolio assessment system for the course “Self-Development and Portfolio,” which is a part of the regular curriculum of the College of Medicine, the Catholic University of Korea. First, we created a list of target competencies for medical students to help students reach the benchmarks that the university requires to graduate. Second, in order to determine the required components of the portfolio, we identified more specific skills that students must master in order to achieve the target competencies for graduation (Supplement 1). Third, we developed evaluation standards for grading performance in portfolio activities through a preliminary implementation and revision, and then finalized specific grading schemes and prepared the corresponding rubrics.

### Content validity test

To verify content validity, we conducted a focus group interview on August 9, 2019. The selected participants were 2 medical education specialists, 2 professors involved in education at the College of Medicine, and a professor of basic medical science. In the focus group interview, candid opinions and feedback were solicited on the portfolio assessment system and essential components of the portfolio, after distributing relevant resources beforehand to ensure that the participants understood the university’s draft of the portfolio assessment system (Supplement 2).

### Rater training process

Ninety-eight portfolios were submitted for the “Self-Development and Portfolio II” course, offered during the second semester of the first year at the College of Medicine in the academic year 2019. Seven portfolios were randomly selected by the course instructor and evaluated according to the assessment system established in step 1. Six professors at the College of Medicine with teaching responsibilities participated as assessors. There were 2 rounds of assessment, followed by assessor training and feedback. During the first training session, we introduced the principles of the portfolio assessment standards, presented their content in detail, and explained the grading system used in the rubric. In the second training session, we attempted to standardize the assessment process by conducting mock grading activities, interpreting each grading standard, and confirming the assessment component represented by each unit of grading. Assessors who completed the training sessions conducted 2 rounds of evaluation for the 7 portfolios.

### Reliability test

We analyzed inter-rater reliability by calculating the inter-class correlation coefficient (ICC), which is appropriate for expressing the reliability of quantitative measurements (Dataset 1). The closer the ICC is to 1.0, the higher the reliability and lower the error variance. If an ICC <0, then the reliability is considered as “poor”; 0–0.20 as “slight”; 0.21–0.40 as “fair”; 0.41–0.60 as “moderate”; 0.61–0.80 as “substantial”; and >0.81 as “almost perfect” reliability [6].

### Statistical methods

The descriptive analysis was done using IBM SPSS ver. 21.0 (IBM Corp., Armonk, NY, USA).

## Results

### Development of the portfolio assessment system

**Fig. 2.**
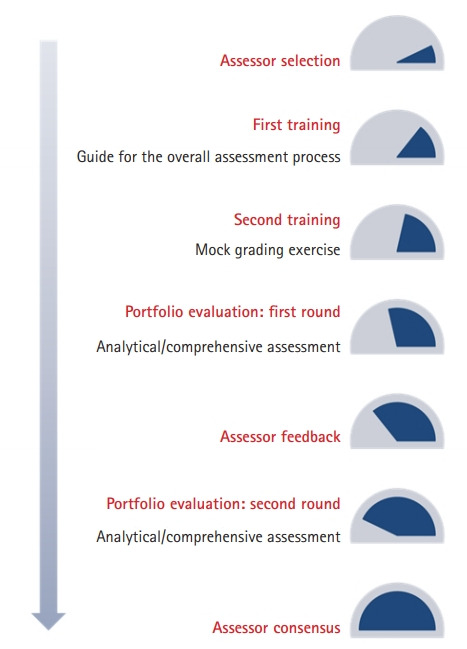
Portfolio assessment system.

We developed a portfolio assessment system that consisted of assessor selection, training, evaluation, and consensus (Fig. 2). The last phase, which involved interpreting and determining the implications of the collected data through consensus, was included to avoid interference from biased or subjective perspectives in the interpretation of students’ performance outcomes during the portfolio evaluation process. The assessment process was divided into analytical assessment and comprehensive assessment. In the analytical assessment, we divided each portfolio into 5 sub-areas and quantified performance on a 5-point scale (excellent, good, average, weak, and poor). In the comprehensive assessment, each portfolio was graded out of 3 points as a whole to facilitate an overall evaluation of each student’s progress. We asked assessors to focus on identifying the student’s learning methods and directions for coaching when writing feedback, rather than on understanding where the student is currently. The final assessment standards, for which content validity was confirmed based on feedback from the expert group after development, along with the grading criteria used for the comprehensive assessment, are presented in Tables 1 and 2. The assessment tool is presented in Supplement 3.

### Contents validity test

The experts agreed that the content validity of the portfolio assessment system was satisfactory. Notably, they predicted that the inclusion of assessor training, a feedback system, and consensus procedures would enable the evaluation to be standardized. The participants in the focus group interviews made the following specific points. First, the mandatory components of the portfolio sufficiently reflected the objective areas that students are recommended to reflect upon and practice during their years in medical school. Second, the assessment standards were designed as practical components where feedback should be provided to evaluate all the performance benchmarks that needed to be assessed based on the learning objectives and outcomes of the “Self-Development and Portfolio” course. Third, the assessment instrument was developed in a way that minimized rating errors by including specific assessment items and grading criteria.

### Reliability test

In the results of the first round of evaluation grades submitted by 6 assessors for 7 randomly selected portfolios, all assessment areas except ‘’goal-setting” showed a high ICC of 0.80 or higher. After the first round of assessment, we attempted to standardize objective assessment procedures through repeated training and mock grading activities. In the analysis of the second round of assessments, the ICCs improved in all areas. Notably, the ICC for “goal setting,” which was 0.769 for the first round of assessments, increased to a high level of reliability (Table 3).

## Discussion

### Key results

The ICC, which is a measure of reliability among multiple assessors, was 0.80 or higher for all assessment areas and all 6 portfolio assessors. This is a meaningful result that confirms the reliability and validity of the portfolio assessment procedure. Of particular note, we enhanced the ICC for the “goal-setting” area by conducting repeated training sessions that included mock grading activities, which confirms the effectiveness of the assessor training component of the assessment system that we developed.

### Interpretation

Portfolio assessment as a method of performance evaluation has a variety of advantages in terms of its inherent intentions and pedagogical interpretation. However, portfolio assessment still faces issues in terms of evaluation, as well as difficulties in practical application in the field. Specifically, it is difficult to guarantee objectivity among evaluators and to ensure the reliability and validity of portfolio assessments. Educational evaluation on portfolio assessment, including analyses of reliability and validity, has been actively conducted in the field of educational evaluation [7]. Similar works have been pursued in the field of health professions education. For example, O’Brien et al. [8] describes the feasibility and outcomes of a longitudinal competency-based electronic portfolio assessment system at a relatively large U.S. medical school. Davis et al. [9] conducted a survey examiner perceptions of Dundee Medical School’s portfolio assessment process, in years 4 and 5 of the 5-year curriculum in the UK medical school. Gadbury-Amyot et al. [10] empirically investigate the validity and reliability of portfolio assessment in 2 U.S. dental schools. Roberts et al. [11] explored the degree to which reliability and validity of a portfolio designed as a programmatic assessment of performance in an integrated clinical placement. Thus, our experience of developing an assessment system to implement in the “Self-Management and Portfolio” course has the following implications. First, specific evaluation standards and a grading rubric should be established to conduct portfolio assessment procedures correctly and appropriately. For this research, we organized a portfolio subcommittee within the curriculum committee to develop the assessment system, including evaluation standards, a grading rubric, and assessment instructions, and we attempted to implement the system methodically. Secondly, prior to the implementation of an assessment system, a training program should be put in place to foster expert assessors. For this study, 2 sessions of assessor training were conducted. We made particular efforts to standardize the assessment standards and grading rubric through the second session, which included mock grading activities and inter-rater feedback. As a result, the ICC, as a measure of inter-rater reliability, increased significantly across all areas (by about 0.02).

### Limitations and generalizability

This assessment system was developed based on the required competencies for graduates of a single medical school. Therefore, it cannot be generalized to all similar institutions. However, we expect this system to become a framework for other medical schools, as it encompasses all basic components of the portfolio assessment system, including a grading rubric and assessment procedures. Moreover, we suggest that follow-up research should be conducted to address the following points. First, it is necessary to verify the educational effects of the portfolio assessment system by dividing subjects into intervention and control groups, and then comparing cognitive and affective variables (e.g., academic achievement, learning motivation, and self-learning capability) between the groups. Secondly, even though we confirmed inter-rater reliability by measuring ICCs, it would also be valuable to assess inter-rater reliability using other methods that would provide more comprehensive information, including generalizability theory as well as the Facets system.

### Conclusion

Through this study, we confirmed that when assessors with an appropriate training conduct portfolio assessment based on specified standards through a systematic procedure, the results are reliable. We also suggested a framework portfolio assessment system that can be used in practice. Although portfolios have been introduced at many medical schools, there needs to be more contemplation regarding the systemic assessment of portfolios. The outcomes of this study are significant, as they suggest the applicability of portfolio assessment in medical education based on methods of ensuring the reliability and validity of portfolio assessment procedures.

## Figures and Tables

**Table 1.** Analytical and comprehensive assessment standards for the portfolio

Type	Assessment area	Assessment items
Analytical assessment	Goal-setting	- Did the student set a worthwhile goal corresponding to the 6 aspects of the educational objectives of training “doctors with a vocational mission,” “capable doctors,” and “doctors with leadership”?
		- Is the goal appropriately challenging and specific enough to be actionable?
	Process	- Has the student engaged in activities and learning appropriate for reaching his or her goal?
		- Were the details of activities and learning described in a specific and concrete manner?
	Reflection	- Did the student reflect on the strengths and weaknesses of his or her learning process and the contents thereof?
		- Did the student reflect on internal aspects of the learning process, not only superficial achievement of the goals?
	Self-study plan	- Did the student’s reflection lead to the identification of specific steps for improvement?
		- Did the student establish specific plans to improve his or her current practices?
	Overall (composition/quality, etc.)	- Did the student comply with the required formatting and successfully organize the materials as a portfolio?
		- Did the student communicate effectively, using appropriate sentence structures and vocabulary?
		- Is the level and quality of relevant learning resources appropriate?
Comprehensive assessment		- Focus on identifying learning practices of the student and directions for future coaching, rather than fact-finding regarding the student’s current situation.

**Table 2.** Grading rubric for comprehensive assessment of the portfolios

Grade	Grading criteria
A	- The student has clear and specific goals for what he or she wants to achieve and learn.
	- The learning strategies and processes to achieve the set goal progressed well in general and the student’s effort is well presented in the portfolio.
	- The portfolio represents the student’s experiences of self-reflection well.
B	- Each component of the portfolio was completed satisfactorily but the content of some components did not include enough specifics or sufficient details.
	- The portfolio conforms to the required format but imperfections can be found in certain components.
C	- The portfolio fails to include at least one basic required component.
	- The student lacks an understanding of the concept of the portfolio and the method of learning.
	- Intensive efforts are needed to enhance the student’s ability to write and utilize a portfolio.

**Table 3.** ICC analysis results

Assessment areas	ICC	
	1st round assessment results	2nd round assessment results
Goal-setting	0.769^**^	0.811^**^
Process	0.852^**^	0.873^**^
Reflection	0.899^**^	0.910^**^
Self-study plan	0.892^**^	0.895^**^
Overall (composition/quality, etc.)	0.887^**^	0.897^**^

ICC, intra-class correlation coefficients.

** P<0.01.

## Footnotes

**Data availability** Data files are available from Harvard Dataverse: https://doi.org/10.7910/DVN/IUEY6B Dataset 1. Raw data of the response from 472 participants.

## Supplementary materials

Data files are available from Harvard Dataverse: https://doi.org/10.7910/DVN/IUEY6B

Supplement 1. Required components for the self-development portfolio.

Supplement 2. Focus group interview form.

Supplement 3. Portfolio assessment form.

Supplement 4. Audio recording of the abstract.
