# Supplementary material for: Development and validation of a portfolio assessment system for medical schools in Korea
Source: J Educ Eval Health Prof. 2020 Dec 9;17:39. doi: 10.3352/jeehp.2020.17.39 (PMC7859386; doi:10.3352/jeehp.2020.17.39)
Supplement: Supplementary file 2 — Supplement 1. Required components for the self-development portfolio. [file jeehp-17-39-suppl1.pdf]

Supplement 1. Required components for the self-development portfolio

**Outline**	Cover, name, student no., etc.
**Self-introduction**	Photo, personal statement, etc.
**Goal setting**	Setting a personal goal during one's time at the school of medicine: focused on 3 educational objectives and 6 areas<br><br>▪ The goal must be worthwhile and challenging for the learner.<br>▪ The goal must be realistic and reachable.<br>▪ The goal must be flexible, so that the learner can adjust it if he or she faces unexpected events or changes.<br>▪ The goal must be specific enough to be actionable.
**Process**	Specific progress of the learning process and details thereof<br><br>▪ The learner selects content to learn and proceeds accordingly, based on his or her personal goals.
**Reflection**	Reflection on learning activities<br><br>▪ After completing the learning activity, the learner reflects on his or her internal self, in addition to the observable achievement of the objectives.
**Self-evaluation on the achievement of goals**	Self-evaluation to determine whether the goal has been accomplished.<br><br>▪ 1 point (poor), 2 points (weak), 3 points (satisfactory), 4 points (sufficient), and 5 points (excellent)
**Self-study plan**	▪ Establish a plan to internalize the learning process and promote further progress. The achievement should not be a one-time event.
**Proof of learning activities and other resources**	▪ Select and gather relevant resources based on the learner's objectives and detailed progression of learning
