# Supplementary material for: Development and validation of a portfolio assessment system for medical schools in Korea
Source: J Educ Eval Health Prof. 2020 Dec 9;17:39. doi: 10.3352/jeehp.2020.17.39 (PMC7859386; doi:10.3352/jeehp.2020.17.39)
Supplement: Supplementary file 3 — Supplement 2. Focus group interview form. [file jeehp-17-39-suppl2.pdf]

Supplement 2. Focus group interview form

포트폴리오 활용 교육 계획(안)

1. 6년 운영 계획

자기돌봄, 자기주도적 평생학습능력 졸업역량 달성 교육

2019년	2020년	2021년	2022년	2023년	2024년
의예1	의예2	의학1	의학2	의학3	의학4
**자기계발과 포트폴리오 I**	**자기계발과 포트폴리오 III**	**임상의학입문 (ICM) I**	**임상의학입문 (ICM) II**	**Core 임상실습**	**Core and Elective 임상실습**
• 나에 대한 이해 대학의 본질과 대학생이 된다는 것의 의미, 대학생으로서의 윤리의식<br>• 성공적인 대학생활과 미래를 위한 마스터플랜 설정<br>• 자기주도적 대학생활: 학습전략, 시간관리 등	• 조기임상 노출 프로그램(early clinical exposure, ECE), 자유쿼터과정 연계 목표 설정<br>• 프로젝트 수행<br>• 프로젝트 결과 발표 등	• 표준화환자면담<br>- 병력청취<br>- 의사소통능력<br>- 태도 등	• 표준화환자면담<br>- 병력청취<br>- 신체진찰<br>- 의사소통능력<br>- 태도 등	• 응급의학 임상 실습(2주)	• 학생인턴십 (4주)
**자기계발과 포트폴리오 II**					
• 도전적 목표 재설정<br>• 포트폴리오 수행<br>• 포트폴리오 경험 나누기<br>• reflective thinking 등					

2. 의예과 1학년 “자기계발과 포트폴리오” 교과목 교육목표 및 학습성과

교육목표
가톨릭 의과대학 신입생으로서<br>첫째, 대학의 교육목적과 목표, 그리고 교육목표에 따른 졸업성과를 이해하고 이를 자신의 대학생활의 목표와 연계 지을 수 있다.<br>둘째, 학습 과정(학과 공부뿐만 아니라 취미나 교양, 심신 단련을 위한 다양한 학습을 포함)에서의 여러 가지 경험을 통해서 자신의 강점과 장점을 파악하는 동시에 자신의 보완하려고 노력하는 과정에서 자신에 대해 더 깊이 성찰하고 이해할 수 있다.<br>셋째, 의예과 2년을 자신의 발전을 위해 좀 더 보람차게 보낼 수 있도록 성찰할 수 있는 기회를 갖는다.<br>넷째, 미래의 의료인으로서 자신의 목표를 달성하기 위해 단계적 계획을 세우고 이를 실현해 가는 과정을 반복함으로써 자기주도적 학습 능력을 함양한다.

학습성과
• 의료인에게 필요한 자기성찰과 자기주도 학습 능력의 중요성을 알고 자질을 계발할 수 있다.<br>• 의미 있는 의예과 생활을 위한 나만의 가치 있는 목표를 수립하고 실천할 수 있다.<br>• 자신만의 효과적인 학습전략을 수립하고 이를 지속적으로 관리하고 발전시킬 수 있다.<br>• 성공적인 학습을 위해서 스스로에게 동기를 부여하며 이를 유지하는 능력과 성공과 실패의 상황에서도 잘 대처하는 능력을 함양할 수 있다.<br>• 포트폴리오를 만들면서 자신의 성취물을 평가하는 능력과 반성적 사고능력을 키울 수 있다.

3. 의예과 1학년 "자기계발과 포트폴리오" 교과목 주요 교육내용

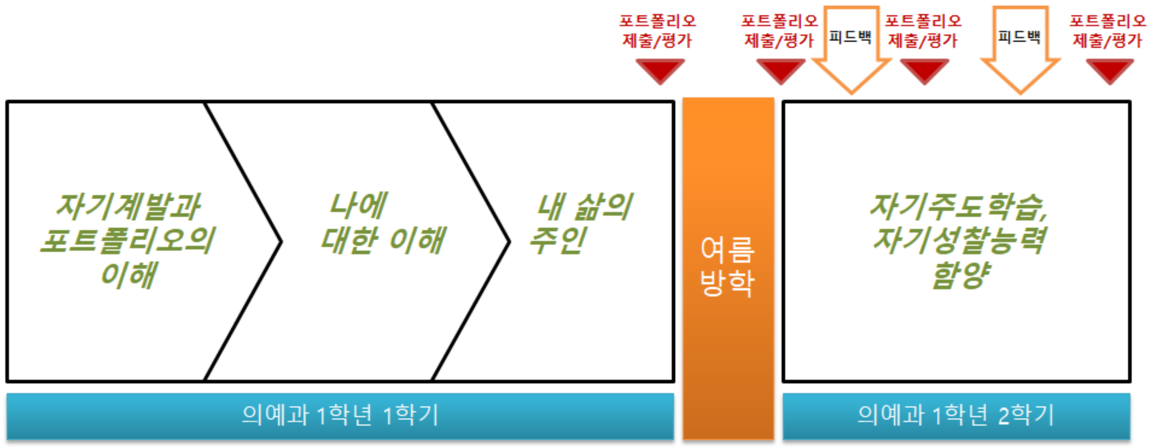

위 교육계획에 근거하여 포트폴리오 필수요소(붙임 1)가 의예과 "자기계발과 포트폴리오" 과정에서 평가해야 내용을 모두 포함하고 있으며, 평가체제(붙임 2)를 통해 이를 제대로 측정할 수 있다고 생각하십니까?

필수구성요소	agree	disagree
평가체제	agree	disagree
