# Supplementary material for: Development and validation of a portfolio assessment system for medical schools in Korea
Source: J Educ Eval Health Prof. 2020 Dec 9;17:39. doi: 10.3352/jeehp.2020.17.39 (PMC7859386; doi:10.3352/jeehp.2020.17.39)
Supplement: Supplementary file 4 — Supplement 3. Portfolio assessment form. [file jeehp-17-39-suppl3.pdf]

Supple 3. Portfolio assessment form

Portfolio no.	1
Assessor	(Signature)

Assessment area	Assessment Items	Assessment (5-point scale)				
		Excellent	Good	Average	Weak	Poor
		5	4	3	2	1
Goal-setting	- Did the student set a worthwhile goal corresponding to the 6 aspects of the educational objectives of training “doctors with a vocational mission,” “capable doctors,’ and “doctors with leadership”?<br>- Is the goal appropriately challenging and specific enough to be actionable?					
Process	- Has the student engaged in activities and learning appropriate for reaching his or her goal?<br>- Were the details of activities and learning described in a specific and concrete manner?					
Reflection	- Did the student reflect on the strengths and weaknesses of his or her learning process and the contents thereof?<br>- Did the student reflect on internal aspects of the learning process, not only superficial achievement of the goals?					
Self-study plan	- Did the student’s reflection lead to the identification of specific steps for improvement?<br>- Did the student establish specific plans to improve his or her current practices?					
Overview	- Did the student comply with the required formatting and successfully organize the materials as a portfolio?<br>- Is the level and quality of relevant learning resources appropriate?<br>- Did the student communicate effectively, using appropriate sentence structures and vocabulary?					

Comprehensive assessment	(Overall rating)	The student completed the portfolio well.	Even though the student satisfactorily prepared each required component of the portfolio, improvement is needed as some components were not specific or sufficient.	The student requires more work due to deficiency in his or her understanding of the concept of the portfolio and learning methods.
		A	B	C
